# Repeating cardiopulmonary health effects in rural North Carolina population during a second large peat wildfire

**DOI:** 10.1186/s12940-016-0093-4

**Published:** 2016-01-27

**Authors:** Melissa A. Tinling, J. Jason West, Wayne E. Cascio, Vasu Kilaru, Ana G. Rappold

**Affiliations:** Department of Horticulture, North Carolina State University, Raleigh, NC 27695 USA; Environmental Sciences and Engineering, Gillings School of Global Public Health, University of North Carolina, Chapel Hill, NC USA; United States Environmental Protection Agency/National Health and Environmental Effects Research Laboratory/Environmental Public Health Division, 109 T.W. Alexander Drive, US EPA, Research Triangle Park, Durham, NC 27707 USA; United States Environmental Protection Agency/National Exposure Research Laboratory/Environmental Sciences Division, Research Triangle Park, Durham, NC USA

**Keywords:** Wildfire smoke, Respiratory effects, Cardiovascular effects, Hypertension, Peat fire

## Abstract

**Background:**

Cardiovascular health effects of fine particulate matter (PM_2.5_) exposure from wildfire smoke are neither definitive nor consistent with PM_2.5_ from other air pollution sources. Non-comparability among wildfire health studies limits research conclusions.

**Methods:**

We examined cardiovascular and respiratory health outcomes related to peat wildfire smoke exposure in a population where strong associations were previously reported for the 2008 Evans Road peat wildfire. We conducted a population-based epidemiologic investigation of associations between daily county-level modeled wildfire PM_2.5_ and cardiopulmonary emergency department (ED) visits during the 2011 Pains Bay wildfire in eastern North Carolina. We estimated changes in the relative risk cumulative over 0–2 lagged days of wildfire PM_2.5_ exposure using a quasi-Poisson regression model adjusted for weather, weekends, and poverty.

**Results:**

Relative risk associated with a 10 μg/m^3^ increase in 24-h PM_2.5_ was significantly elevated in adults for respiratory/other chest symptoms 1.06 (1.00–1.13), upper respiratory infections 1.13 (1.05–1.22), hypertension 1.05 (1.00–1.09) and ‘all-cause’ cardiac outcomes 1.06 (1.00–1.13) and in youth for respiratory/other chest symptoms 1.18 (1.06–1.33), upper respiratory infections 1.14 (1.04–1.24) and ‘all-cause’ respiratory conditions 1.09 (1.01–1.17).

**Conclusions:**

Our results replicate evidence for increased risk of cardiovascular outcomes from wildfire PM_2.5_ and suggest that cardiovascular health should be considered when evaluating the public health burden of wildfire smoke.

**Electronic supplementary material:**

The online version of this article (doi:10.1186/s12940-016-0093-4) contains supplementary material, which is available to authorized users.

## Background

Air pollution is recognized as a major global health burden [1]. Human health impacts of air pollution range from short-term effects to chronic diseases, and are primarily related to the cardiovascular and respiratory systems [2]. In the past several decades numerous educational and outreach programs have been established to reduce the health burden while policies to curb air pollution have effectively reduced anthropogenic emissions. However, emissions from some naturally occurring processes such as wildfires are on the rise globally. Many questions remain in our understanding of how to protect the health of the most vulnerable populations in the face of wildfire air pollution.

The development of effective policies to protect public health during wildfire episodes requires characterizing the specific health effects of smoke exposures and identifying the subpopulations that are most susceptible. Of the mixture of pollutants people are exposed to through wildfire smoke, fine particulate matter (PM_2.5_) poses the greatest health concern. Current epidemiologic evidence of health effects observed from exposure to wildfire PM_2.5_ is not consistent with that of PM_2.5_ exposure from other air pollution sources [3, 4]. In particular, while PM_2.5_ exposure from urban sources has been determined “causal” for cardiovascular health outcomes, the evidence for wildfire PM_2.5_ is not definitive [5]. This raises the question of whether the causal relationships of urban PM_2.5_ exposure to cardiovascular disease can be generalized to wildfire PM_2.5_.

The evidence leading to causal associations between PM_2.5_ in urban environments and cardiovascular morbidity and mortality is based on clinical and epidemiological evidence of more than 1000 studies conducted over several decades. By contrast, there are significantly fewer studies of wildfire PM_2.5_ cardiovascular effects [6–11] and fewer still focused on peat wildfires [10]. The unpredictable nature of wildfire events limits prospective research design. Studies are constrained by the retrospective availability of health and exposure data and vary widely with respect to population characteristics, health indicators, exposure duration, and exposure assessment. Additionally, meta-analysis has never been conducted due to insufficient number of studies with complementary designs.

In their study of the 2008 Evans Road wildfire in the Pocosin Lakes National Wildlife Refuge in eastern North Carolina (NC), Rappold et al. [10] reported strong associations of smoke exposure and emergency department visits for various respiratory syndromes and congestive heart failure. The results of their study raised concerns that the cardiovascular effects of wildfire smoke exposure had not been adequately captured in previous literature. In 2011 a wildfire of similar size ignited in the Pains Bay area of the Alligator River National Wildlife Refuge in neighboring Dare County, NC, several miles away from the 2008 site. Much as in 2008, sustained drought conditions facilitated smoldering combustion of the deep organic peat soils, producing dense smoke plumes that blanketed the region on several days. More than 5000 acres of pocosin forest and $14.5 million in firefighting resources were consumed before Hurricane Irene extinguished the blaze and residual smoldering in late August 2011 [12].

The objective of this study is to reexamine the impact of peat wildfire smoke exposure on cardiovascular and respiratory outcomes in a population where such associations were previously observed under similar conditions. We use health outcome data from the same statewide emergency department syndromic surveillance program as Rappold et al. [10]; however, here we use PM_2.5_ concentration estimates from smoke plume dispersion modeling instead of satellite measured aerosol optical depth. The re-occurrence of peat wildfire in the same population provides a unique opportunity to determine the reproducibility of the health associations between peat wildfire smoke and cardiopulmonary outcomes. Results from this research will assist public health professionals in generalizing health risks of wildfire smoke exposure and raising awareness of population vulnerability.

## Methods

### Study area and period

The study area included 28 contiguous counties in eastern NC selected for analysis from the larger coastal region which experienced at least one 24-h average smoke-attributable PM_2.5_ concentration exceeding 20 μg/m^3^ during the study period, as estimated by smoke plume dispersion modeling. The 28 study counties were largely rural but varied in socioeconomic characteristics. For example, the percent of county population in poverty (all ages) ranged from 8.5 % in Currituck County to 30.2 % in Robeson County (median 17.7 %, interquartile range 15.3–22.1 %; 2010) [13]. The total population of the study area was 1,841,372, of which 24.0 % were less than 18 years old and 12.5 % were over 65 [13].

The study period consisted of 45 days between the ignition of the Pains Bay fire on May 5^th^, 2011 and that of an independent wildfire in Pender County, NC on June 19^th^, 2011. Although the Pains Bay fire continued to burn through August, days beyond June 18^th^ were excluded from this study to eliminate potentially overlapping smoke exposures. Unlike the 2008 episode, this study period coincided with unusually warm temperatures and large variation in humidity. The temperature in Greenville, NC reached 100 °F on May 31^st^, breaking the record in NC for the earliest date reaching this mark [14]. Daily average relative humidity in the study area ranged from 54.7 to 79.8 % with an average of 67.8 % over the study period [14].

### Syndromic surveillance

We used the same health outcome data source as was used by Rappold et al. [10]: the North Carolina Disease Event Tracking and Epidemiologic Collection Tool (NC DETECT) [15]. NCDETECT is a uniquely comprehensive statewide syndromic surveillance program, with near universal coverage of NC hospitals’ Emergency Departments with up to 11 discharge ICD-9-CM codes (International Statistical Classification of Disease, 9^th^ revision, Clinical Modification (ICD-9-CM) [16]).

We obtained de-identified records of emergency department (ED) visits for respiratory and cardiac related outcomes hypothesized to be affected by smoke. Each record contained subject age, gender, date of visit, county of residence, and as many as eleven assigned ICD-9-CM discharge codes. We considered all discharge codes related to each ED visit and aggregated them into eight outcome groups previously considered in Rappold et al. [10]. The eight outcome groups included discharge codes related to asthma (493), chronic pulmonary conditions (490, 491, 492, 496), acute respiratory infections (466, 481, 482, 485), heart failure (428), cardiac dysrhythmia (427), respiratory/other chest symptoms (786), pooled “all-cause” respiratory conditions (460:466, 480:486, 490:493, 496), and pooled “all-cause” cardiac conditions (410, 411, 413, 415, 416, 417, 420:429, 434, 435, 444, 445, 451) (Table 1). In addition to these, we also included hypertension (401) as an important clinical condition leading to heart failure. Bone fractures (800–849) was a control outcome group hypothesized not to be affected by smoke. Outcomes with low daily counts, such as stroke and chronic bronchitis, were included in “all-cause” pooled groups for cardiac and respiratory conditions respectively. Because of lack of specificity, respiratory/other chest symptoms (786) describing patients presenting symptoms such as chest pain and labored breathing, were not pooled into either respiratory or cardiac “all-cause” outcome groups but rather considered as a separate outcome group. Aggregated counts and the list of discharge codes for each outcome are given in Table 1.

**Table 1:** Frequency of ED discharge codes by age and gender

	Youth (<18)	Mid-age^a^ (18–64)	Elderly (65+)	All adults^b^			Total^c^
				F	M	All	
Respiratory/other chest symptoms (786)	2,338	11,246	3,449	8,586	6,108	14,695	17,033
All-cause respiratory^d^	5,562	10,678	3,644	9,198	5,123	14,322	19,884
Asthma (493)	1,939	3,718	-^e^	3,111	1,201	4,312	6,251
Chronic pulmonary conditions^f^	-	2,726	1,905	2,621	2,009	4,631	5,161
Upper respiratory infections^g^	2,794	3,428	-	2,551	1,112	3,663	6,457
All-cause cardiac^h^	-	4,438	8,313	6,820	5,931	12,751	12,751
Cardiac dysrhythmia (427)	-	1,269	2,712	2,126	1,855	3,981	3,981
Heart failure (428)	-	1,604	3,114	2,636	2,082	4,718	4,718
Hypertension (401)	-	4,186	3,796	4,750	3,232	7,982	7,982
Total	7,900	30,548	19,202	29,354	20,394	49,750	57,650

^a^ Minimum age for “Mid-age” and “All adults” is 18 years for Respiratory/other chest symptoms and Respiratory diagnoses, and 45 years for Cardiac diagnoses and Hypertension

^b^ “All adults” includes “Mid-age” and “Elderly”

^c “^Total” includes “All adults” and “Youth” (where applicable)

^d^ All-cause respiratory ICD-9-CM codes: 460:466, 480:486, 490:493, 496

^e^ This symbol (-) represents a subset in which counts were not high enough to afford statistical analysis

^f^ ICD-9-CM codes 490, 491, 492, 496

^g^ ICD-9-CM codes 460–465

^h^ All-cause cardiac ICD-9-CM-CM codes: 410, 411, 413, 415, 416, 417, 420:429, 434, 435, 444, 445, 451

The resulting daily counts for each outcome group were aggregated by county of residence for each of the 28 exposed counties and stratified by age and gender for statistical analysis. Cardiac outcomes and hypertension were stratified for mid-aged adults (45–64), elderly (65+), and all adults (45+) based on previous reports. Age strata for respiratory conditions and respiratory/other chest symptoms were mid-aged adults (18–64), elderly (65+), and all adults (18+). Youth (<18) were analyzed for asthma, upper respiratory infections, respiratory/other chest symptoms and pooled “all-cause” respiratory conditions. Counts of youth and elderly in other outcomes were too low to be statistically informative. Gender stratification was performed for the all-adult age groups. The Institutional Review Board at the University of North Carolina, Chapel Hill approved the study protocol.

### Exposure estimate

**Fig. 1:**
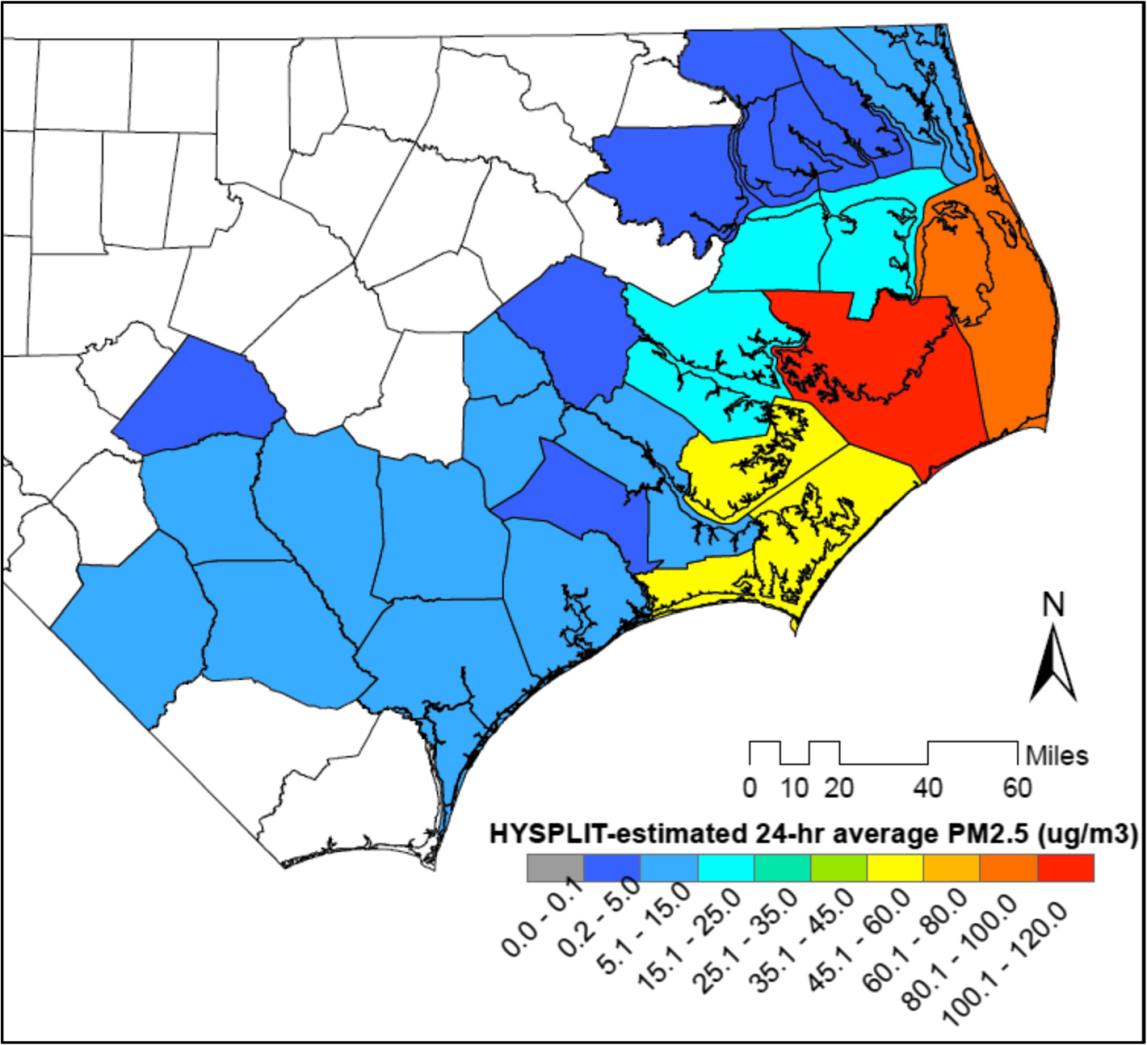
Average 24-h wildfire PM_2.5_ exposure estimate, 28 study counties in eastern NC: May 12, 2011

We approximated county-level, daily exposures to wildfire PM_2.5_ (μg/m^3^) using modeled predictions from the Smoke Forecasting System (SFS). The SFS is developed and managed by the National Air Resources Laboratory (ARL) of the National Oceanic and Atmospheric Administration (NOAA) [17] and provides hourly wildfire smoke forecasts and reanalysis for the entire US since 2007 [18]. The SFS uses the US Forest Service’s BlueSky Framework for wildfire emissions and the Hybrid Single Particle Lagrangian Integrated Transport model (HYPSLIT) to calculate air parcel trajectories and to simulate dispersion and deposition of wildfire-generated air pollutants [19]. Concentrations of fine particulate matter (PM_2.5_) from other sources are not modeled by SFS. For the analysis, we calculated 24 h averages (midnight-to-midnight) for each grid point and averaged daily concentrations across the counties (Fig. 1). Any 24-h county estimate less than 0.1 μg/m^3^ was considered below the detection limit and replaced with (0.1/√2 μg/m^3^) [20].

### Exposure summary

**Fig. 2:**
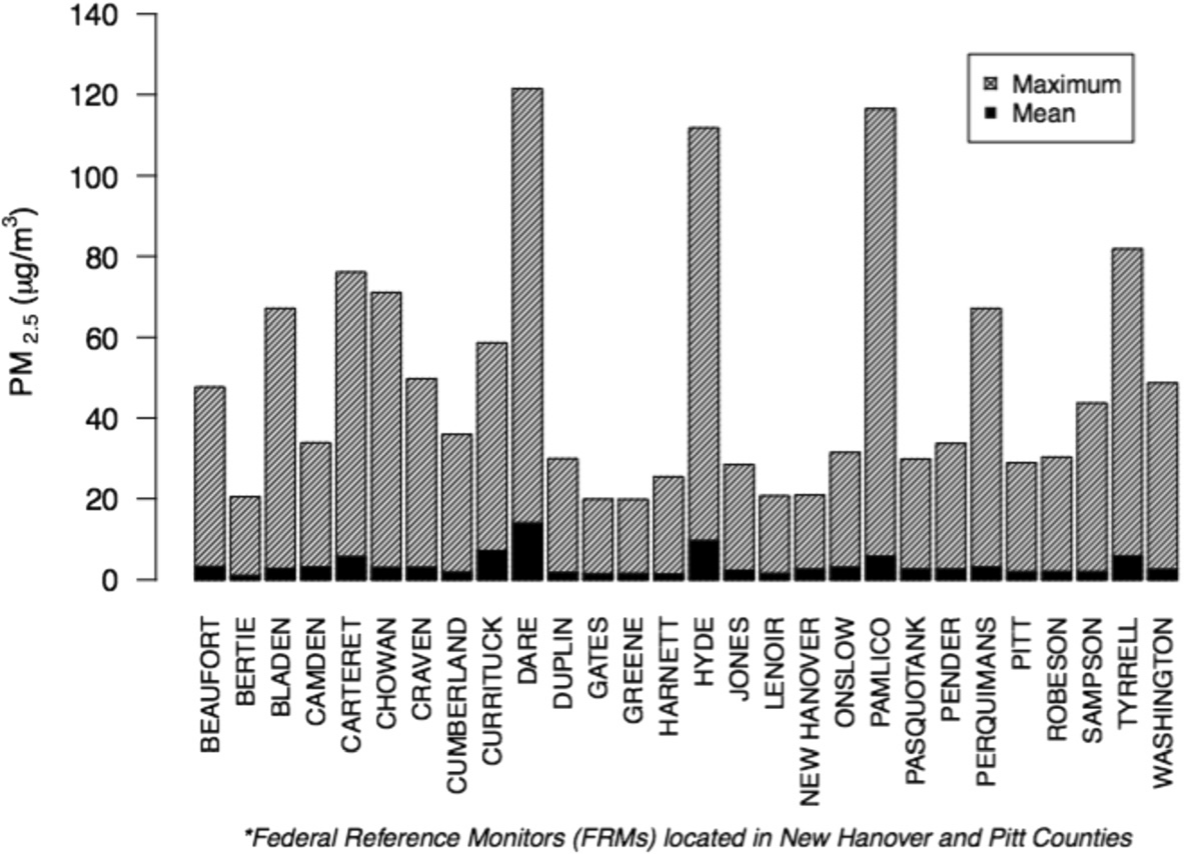
County daily maximum and average wildfire PM_2.5_ concentrations May 5^th^- June 18^th^, 2011

SFS estimations of wildfire PM_2.5_ varied greatly by county and day, reflecting the unpredictable and episodic nature of wildfire smoke. The three highest 24-h county average concentrations all exceeded 100 μg/m^3^ and were clustered over the same three-day period in early May: Pamlico County on May 11^th^ (116.5 μg/m^3^), Hyde County on May 12^th^ (111.9 μg/m^3^) and Dare County on May 13^th^ (121.4 μg/m^3^). Other peak exposure-days common among several counties occurred on May 22^nd^, May 31^st^, June 7^th^ and June 16^th^; however, there was no sustained multi-day period of severe smoke exposure affecting a broad swathe of counties as occurred in 2008. Overall, Dare and Pamlico counties experienced the highest burden of wildfire PM_2.5_ during the study period (Fig. 2).

We examined temporal relationships between SFS estimates of wildfire PM_2.5_ concentrations and measured concentrations at five ground monitors in the region (Additional file 1: Figure S2) [21]. The five monitors included three emergency monitors in Manteo, Greenville and Washington, NC temporarily set up in response to high exposures experienced by these three cities, as well as two federal reference PM_2.5_ monitors located in New Hanover and Pitt counties (US EPA). We compared concurrent 24-h PM_2.5_ concentrations measured by monitors with SFS predictions averaged within a ten-mile radius of each monitoring site using linear regression [22]. The modeled smoke plume shape and direction agreed well with satellite imagery; however, the association of SFS and ground monitors was weak. The Manteo emergency monitor agreed most closely with SFS (coefficient of determination, R^2^ = 0.30). This site was the closest to the actual fire at approximately 20 miles to the northeast and operated continuously from May 26^th^ through the end of the study period. Although the concentrations differed, most of the days when the Manteo monitor detected elevated PM_2.5_ were also estimated by SFS to have had smoke presence. Agreement was weakest at the Greenville site located approximately 85 miles west of the fire (R^2^ = 0.05). Data from both sources suggests that Greenville did not experience substantial wildfire smoke exposure on monitor measurement days. Finally, between the two federal reference monitors the New Hanover site agreed more closely with SFS (R^2^ = 0.26) than Pitt County (R^2^ = 0.05); however, the Pitt site only monitored every three days. Even though background air pollution in the region accounts for only a fraction of smoke-related PM_2.5,_ strong agreement could not be expected between SFS predictions which do not measure background levels and monitored measurements which do. For adequate comparisons a longer time series of available data and finer special resolution is needed.

### Statistical analysis

We conducted a population-based epidemiological analysis to examine county-level associations between daily wildfire PM_2.5_ and ED visits (Y_ct,_) for each health outcome separately using a quasi-Poisson regression model with logged county population (Z_c_) as a baseline rate offset (Equation 1). Associations for each of the nine diagnosis groups were additionally assessed for selected age and gender strata using corresponding log-transformed population size (Zc). We summarized the cumulative effect over exposure lag days 0, 1, and 2 to account for biologically plausible delays in health effects following PM_2.5_ exposure. The relative risk over lag days 0–2 was estimated using an unconstrained distributed model and the cumulative effect (cRR) was expressed per 10 μg/m^3^ increase in wildfire PM_2.5_ (Equation 2). The statistical model also included indicator variables for county-level meteorological conditions obtained from the North Carolina Climate Retrieval and Observations Network of the Southeast [14]. More specifically, for each exposure day we calculated the average temperature and relative humidity of the previous three days $\left\{\left(\overline{RH_{1-3}}\right),,,\left(\overline{{\mathrm{T}}_{1-3}}\right)\right\}$, as well as the change between that average and the current day’s conditions to account for sudden fluctuations {(RH_0_ - RH_1-3_), (T_0_ - T_1-3_)}. We accounted for baseline risk variation due to outdoor activity levels using indicator variables for weekends and the Memorial Day holiday (May 27–30, 2011). Finally, we controlled for county-level socioeconomic status (SES) variability using the percent of county population in poverty (Additional file 1: Figure S1) [13]. Estimated changes in relative risk were determined statistically significant if *p*-values were smaller than 0.05 and borderline significant if *p*-values were greater or equal than 0.05 but smaller than 0.1. All statistical analysis was conducted using the R Project for Statistical Computing [23].

1 $$ log\left(E\left({Y}_{ct}\right)\right)=\left\{\begin{array}{l}{\alpha}_c+{\beta}_0P{M}_{ct}^{lag0}+{\beta}_1P{M}_{ct}^{lag1}+{\beta}_2P{M}_{ct}^{lag2}\\ {}{\beta}_3\overline{T_{ct}^{1-3}}+{\beta}_4\left({T}_{ct}^0-\overline{T_{ct}^{1-3}}\right)+{\beta}_5\overline{R{H}_{ct}^{1-3}}+{\beta}_6\left(R{H}_{ct}^0-\overline{R{H}_{ct}^{1-3}}\right)+\\ {}{\beta}_7{1}_{Weeken{d}_t}+{\beta}_8{1}_{MemorialDayWeeken{d}_t}+\\ {}{\beta}_9 Povert{y}_c+{Z}_c\end{array}\right. $$

2 $$ cRR= \exp \left\{{\displaystyle {\sum}_{i=0}^2{\beta}_i\times 10}\right\} $$

## Results

**Fig. 3:**
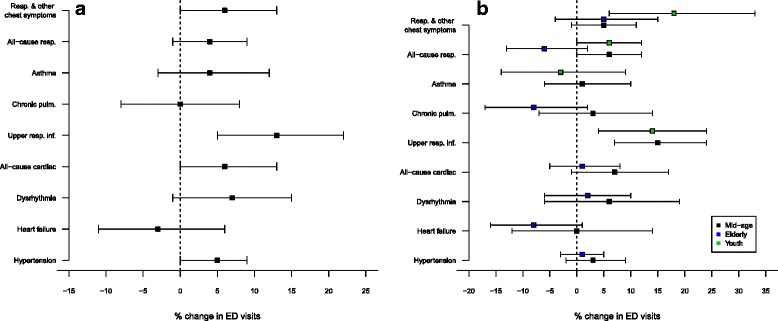
Percent change in relative risk (cumulative lag days 0–2) and 95 % confidence intervals per 10 μg/m^3^ rise in wildfire PM_2.5_ by outcome for (**a**) all adults, (**b**) by age group. Only outcomes with sufficient counts are given (see Table 1)

Emergency department (ED) visits related to cardiac and respiratory conditions were significantly associated with wildfire PM_2.5_ exposure for all age groups (Table 2). Among adults, we observed significantly increased risk of ED visits related to upper respiratory infections (*cRR* = 1.13 [95 % *CI* = 1.05–1.22]), all-cause cardiac conditions (1.06 [1.00–1.13]), hypertension (1.05 [1.00–1.09]) and respiratory/other chest symptoms (1.06 [1.00–1.13]) and borderline significant increases for cardiac dysrhythmia related visits (1.07 [0.99–1.15]). Relative risk (cRR) was also elevated for all-cause respiratory and asthma related visits, though not statistically significant. No associations were observed for bone fractures. Figure 3a displays the cumulative relative risk (cRR) over lag days 0–2 and the corresponding 95 % confidence intervals for adults in each ED outcome group associated with a 10 μg/m^3^ increase in exposure to wildfire PM_2.5_.

**Table 2:** Cumulative relative risk and 95 % confidence intervals associated with a 10 μg/m^3^ increase in PM_2.5_ concentration for all outcome groups, all age and gender strata

ED visits related to^a^	Age/gender strata^b^	cRR	Lower 95 % CI	Upper 95 % CI	*p*-value & significance^c^	
Respiratory/other chest symptoms	Youth	1.18	1.06	1.33	<0.01	**
	Mid-age	1.05	0.99	1.11	0.13	
	Elderly	1.05	0.96	1.15	0.26	
	All adults	1.06	1.00	1.13	0.05	*
	Female all adults	1.05	0.98	1.13	0.14	
	Male all adults	1.09	1.01	1.18	0.03	*
All-cause respiratory conditions	Youth	1.09	1.01	1.17	0.03	*
	Mid-age	1.06	1.00	1.12	0.04	*
	Elderly	0.94	0.87	1.02	0.15	
	All adults	1.04	0.99	1.09	0.13	
	Female all adults	1.06	1.00	1.12	0.05	°
	Male all adults	1.03	0.96	1.11	0.45	
Asthma	Youth	0.97	0.86	1.09	0.60	
	Mid-age	1.01	0.94	1.10	0.72	
	All adults	1.04	0.97	1.12	0.29	
	Female all adults	1.02	0.94	1.11	0.61	
	Male all adults	1.11	0.99	1.25	0.07	°
Chronic pulmonary conditions	Mid-age	1.03	0.93	1.14	0.53	
	Elderly	0.92	0.83	1.02	0.12	
	All adults	1.00	0.92	1.08	1.00	
	Female all adults	1.05	0.95	1.15	0.36	
	Male all adults	0.96	0.85	1.08	0.47	
Upper respiratory infections	Youth	1.14	1.04	1.24	<0.01	**
	Mid-age	1.15	1.07	1.24	<0.01	***
	Elderly	0.64	0.39	1.05	0.08	°
	All adults	1.13	1.05	1.22	<0.01	**
	Female all adults	1.14	1.05	1.24	<0.01	**
	Male all adults	1.13	0.99	1.29	0.07	°
All-cause cardiac conditions	Mid-age	1.07	0.99	1.17	0.09	°
	Elderly	1.01	0.95	1.08	0.71	
	All adults	1.06	1.00	1.13	0.04	*
	Female all adults	1.10	1.02	1.18	0.01	*
	Male all adults	1.03	0.95	1.12	0.42	
Cardiac dysrhythmia	Mid-age	1.06	0.94	1.19	0.37	
	Elderly	1.02	0.94	1.10	0.62	
	All adults	1.07	0.99	1.15	0.07	°
	Female all adults	1.08	0.98	1.19	0.12	
	Male all adults	1.06	0.96	1.18	0.26	
Heart failure	Mid-age	1.00	0.88	1.14	0.99	
	Elderly	0.92	0.84	1.01	0.09	
	All adults	0.97	0.89	1.06	0.50	
	Female all adults	1.01	0.91	1.12	0.87	
	Male all adults	0.93	0.82	1.06	0.29	
Hypertension	Mid-age	1.03	0.98	1.09	0.19	
	Elderly	1.01	0.97	1.05	0.62	
	All adults	1.05	1.0	1.09	0.04	*
	Female all adults	1.07	1.02	1.13	<0.01	**
	Male all adults	1.03	0.97	1.09	0.36	
Bone fractures	Youth	0.98	0.87	1.11	0.74	
	Mid-age	0.97	0.91	1.04	0.45	
	Elderly	1.01	0.89	1.15	0.90	
	All adults	1.06	0.97	1.15	0.20	
	Female all adults	1.00	0.93	1.08	0.99	
	Male all adults	1.03	0.9	1.19	0.67	

^a^ See Table 1 for listings of ICD-9-CM codes included in each diagnosis group

^b^ See Table 1 for listing of ages included in each age group

^c^ Symbols refer to the level of significance: [°] 0.05 ≤ *p* < 0.1, [*] *p* < 0.05, [**] *p* < 0.01, [***] *p* < 0.001

Daily ED visits in all-adult females were elevated for all outcome groups; however, only following were significant: all-cause cardiac conditions (1.10 [1.02–1.18]), hypertension (1.07 [1.02–1.13]), and upper respiratory infections (1.14 [1.05–1.24]) (Additional file 1: Figure S3). A borderline significant increase in risk was found for all-cause respiratory outcomes (1.06 [1.00–1.12]. For male adults, cRR was elevated for all outcomes except those related to chronic pulmonary conditions and heart failure. A significant increase in risk was found for respiratory/other chest symptoms (1.09 [1.01–1.18]) and a borderline significant increase in risk was found for ED visits related to asthma (1.11 [0.99–1.25]) and upper respiratory infections (1.13 [0.99–1.29]).

The highest increases in cRR were observed for youth strata (Fig. 3c). The risk of respiratory/other chest symptoms increased 18 % per 10 μg/m^3^ increase in exposure over 0–2 lag days (1.18 [1.06–1.33]). Youth cRR increases were also significant for upper respiratory infections (1.14 [1.04–1.24]) and all-cause respiratory conditions (1.09 [1.01–1.17]). For mid-age adults, we observed significant increases in cRR for all-cause respiratory conditions (1.06 [1.00–1.12]) and upper respiratory infections (1.15 [1.07–1.24]). In the same strata we also observed a borderline significant increase in daily rates of all-cause cardiac outcomes (1.07 [0.99–1.17]). The risk for respiratory/other chest symptoms, hypertension, cardiac dysrhythmia, chronic pulmonary conditions and asthma were also increased but not statistically significant. We did not observe significant changes in risk for any ED outcomes examined in the elderly strata.

### Poverty

County-level poverty was found to be a confounding variable for all outcomes. This indicator of socioeconomic status (SES) was a significant independent predictor of ED visits during the study period while also independently associated with smoke exposure. Counties with the highest estimated wildfire PM_2.5_ concentrations had lower percentages of population living in poverty compared to less-exposed counties, thus confounding the results. In other words, counties with higher rates of poverty were less impacted by smoke than wealthier counties. Estimates of relative risk from a crude regression model unadjusted for poverty were consistently lower than for the regression model we selected that did adjust for poverty (Additional file 1: Figure S4). ANOVA tests used to compare deviance between statistical models controlling for and not controlling for poverty indicated that including this adjustment greatly improved the regression model fit. We conducted an additional regression analysis unadjusted for SES on a subset of the eleven most highly exposed counties and found cRR estimates consistent with those from the adjusted regression in the full 28-county study area (data not shown), thus providing further evidence of confounding by poverty in the 28-county analysis. We conclude that population SES confounded statistical relationships between smoke exposure and health outcomes in this 2011 peat wildfire.

## Discussion

### Comparison to 2008 Evans road wildfire

Comparing health impacts of wildfire events across world regions is difficult due to variation in combustion intensity, fuel type, population, and other factors. Peat wildfires also differ from hardwood fires because of the smoldering low-oxygen combustion of peat fuels. Our research provides a unique opportunity to compare health risks of peat fire smoke exposure with that of another fire which shared the same ecology, at-risk population, and health outcome definition. The overall changes is risk of cardiopulmonary ED visits during this 2011 fire were similar in nature but lower in magnitude than those reported for the 2008 fire. We found increased risk of cardiac and respiratory related ED visits associated with wildfire PM_2.5_ exposure, specifically for adult upper respiratory infections, respiratory/other chest symptoms, hypertension, and cardiac outcomes. In comparison, Rappold et al. [10] found significant associations for asthma, COPD, pneumonia, congestive heart failure and chest pain symptoms, as well as borderline significant associations for upper respiratory infections and cardiovascular outcomes. While both studies observed statistically significant increased risk for respiratory conditions, upper respiratory infections and respiratory/other chest symptoms, for the 2011 fire the increases in risk of asthma related ED visits were less conclusive and the increases in risk of cardiovascular outcomes were more conclusive.

There are several possible explanations for the overall weaker associations observed in this study compared to 2008 fire. First, PM_2.5_ concentrations in 2011 were generally lower than those in 2008 and more intermittent. The 2008 fire caused a sustained three-day severe smoke episode across most of eastern NC, while smoke presence in 2011 was intermittent without extended periods of such severe and widespread plumes. To reduce possible confounding induced at longer lag days by intermittent exposures we summarized cumulative effects over a shorter time period (lag days 0–2 instead of lag days 0–5 used for the 2008 fire). By summarizing the effects over shorter time period we may have underestimated some effects. The three-day episode in 2008 allowed for comparison of ED visits before and after the exposure period for a large group of combined counties, possibly increasing the power of statistical analysis, whereas in the current analysis we classified exposure by county-day. The reoccurrence of wildfire near Pains Bay, NC within a three-year period also may have improved population awareness; therefore, it is possible that asthmatics and health-compromised persons may have modified their behavior in 2011 to make emergency care unnecessary [24, 25]. Improved health care access and quality in the region since 2008 may also have reduced health impacts. Finally, the 2008 wildfire occurred June to July while the 2011 fire began during an unseasonably warm May, possibly implicating differences in environmental conditions.

### Cardiovascular health impact

The results provide supporting evidence that cardiovascular health should be considered when evaluating the public health burden of wildfire smoke. We found significantly elevated cRR in adults for all-cause cardiac conditions, cardiac dysrhythmia, hypertension and respiratory/other symptoms. Some studies have found no significant associations between wildfire smoke exposure and cardiac outcomes [26–29]. Delfino et al. [7] found small increases for cardiac conditions (*RR* = 1.01 [95 % *CI* = 0.99–1.02]); however, they found slight inverse associations for dysrhythmia whereas we found significant positive increases in cRR (lag days 0–2) for all-cause cardiac and for cardiac dysrhythmia. Fewer and more recent studies found stronger associations with cardiovascular outcomes [8–11, 30].

One interesting finding in this study was the significant increase in cRR for adult hypertension. Arbex et al. [30] reported increased incidence of hypertension hospital admissions associated with burning agricultural biomass in Argentina. A review by Brook and Rajagopalan [31] suggested that individuals with pre-existing hypertension might be more susceptible to elevated blood pressure following particulate exposure. Stress induced by living with sustained smoke and interruptions to daily activities caused by a wildfire may also influence hypertension. Elevated blood pressure may be an important mechanism in the development of cardiovascular health effects from exposure. During the study period, 3821 ED visit records for adults over the age of 45 had a discharge code for both hypertension and a cardiac condition in the “all-cause cardiac” outcome group. To rephrase, about one third of the adult ED visits in our sample that were discharged with a cardiac code were also coded for hypertension. Therefore, the increase in hypertension risk in this study and the co-occurrence of hypertension and cardiac diagnoses suggests blood pressure elevation may have played a role in the observed effects. This research study provides additional evidence that increased cardiac morbidity can be associated with wildfire PM_2.5_ exposure in certain populations.

### Limitations

Exposure misclassification is a likely limitation of our study. We characterized exposure based on county of residence leading to possible misclassification or omission of individuals who spent a significant portion of their time elsewhere. Such misclassification would likely bias our results toward the null hypothesis. Individuals residing in the same county may also experience different exposures depending on lifestyle factors such as occupation, recreation, housing quality and air conditioning use, as well as individual protective behavior modifications. The low daily incidence rate of specific health outcomes in subpopulations, such as asthma and upper respiratory infections in the elderly population, was another limitation to our research. The elderly population is commonly viewed as one of the most susceptible populations but we did not have adequate daily counts to assess the associated health risks in this research. They also tend to be well informed about environmental factors that impact their health and more likely to modify their behavior.

We found limitations in directly comparing observed associations between health outcomes and smoke due to differences in exposure during the two fires as well. First, the environmental and climatic factors made the smoke production during two fires quite different leading us to use different exposure metrics. Unique environmental conditions during the 2008 fire allowed for the use of satellite AOD and defining ‘exposed’ and ‘unexposed’ counties during a clearly defined 3-day smoke episode; however, in 2011 the smoke production was substantially more intermittent and extended over a longer period of time. Additionally, without a clear ‘cut off’ value for AOD classification of exposure, averaging of AOD to county-day would likely have led to additional misclassification error. Additionally, during rapidly varying wind conditions such as in 2011, satellite measured AOD may not realistically represent ground level exposures. Second, even though two fires ignited only several miles apart, the wind patterns during two events were very different inducing dissimilar spatial distribution of exposure within the region. During the 2008 fire 18 counties were affected by sustained smoke levels while in 2011 27 counties were affected intermittently. While most of the counties were impacted by both fires, some were not, and exposure levels varied.

Unlike ground-level air quality monitors, SFS only estimates the PM_2.5_ attributable to wildfire emissions, thereby giving an incomplete picture of the overall population PM_2.5_ exposure. For example, urban areas that were not as affected by smoke may have still experienced elevated PM_2.5_ on certain days, further biasing the results towards the null. In the future we expect air monitoring will become more comprehensive, allowing for more accurate PM_2.5_ estimates and easier cross-referencing with other exposure sources [26].

Investigation of ED visits at the county level likely under-represents the total public health burden of a wildfire event. Many individuals experiencing symptoms will not visit the ED. Alternate healthcare transactions such as visiting a doctor are not captured by NC DETECT. Conversely, ED physicians may have higher diagnostic suspicion during wildfire health events, potentially leading to greater overall diagnoses. More studies are needed to incorporate additional metrics of lower-severity health outcomes such as physician visits, over-the-counter medication sales, or internet search trends to better capture total public health impact of wildfire smoke.

### Public health relevance

The annual US acreage burned by wildfires has tripled over the last 30 years and the number of wildfires now exceeds 70,000 per year. While most wildfires occur in isolated areas with small populations, a growing number are impacting urban populations. Federal, state and local fire and public health officials are challenged to provide effective population advisories. Rappold et al. [32] found that interventions based on smoke forecasts can reduce the economic and public heath burden of wildfires. While evacuation orders are generally given when life or property is immediately threatened, public health advisories to limit exposure are more problematic because the trigger point for taking action is not well defined. This uncertainty is in part related to the paucity of health outcome data related to wildfire smoke exposure and limitations in predicting smoke plumes due to changing weather patterns. The results here show consistent health effects from the emissions of burning peat that can be used in estimating the impacts across a population and help guide the development of public health advisories.

Air pollution emitted by peat fires presents a unique concern to global climate and health. Unlike other wildfires, fires in peat soil smolder at lower temperatures leading to less efficient combustion over a longer period of time. As a result, peat fires produce dense ground level plumes with high concentrations of volatile organics that may be more harmful to health than smoke from hardwood forest fires. In addition, peatland fires emit massive amounts of greenhouse gases trapped in soil [33, 34] particularly carbon and methane, further contributing to climate change. A recent report by the World Resources Institute [35] estimated that peat fires have a 200 times greater impact on global climate change then fires on other lands combined. The report estimates that the emissions from 100,000 detected peat fires in Indonesia during the summer of 2015, exceeded daily emissions of entire US economy on at least 20 days. Smoke from these and other peatland fires are reported to regularly shut down commercial traffic, schools and public events in the region while overwhelming hospitals with excess of respiratory tract infections and other haze-related illnesses [36–38]. In the US peat fires are less common but are increasing in frequency in part due to extended periods of drought and past land use practices. Most peatlands are found in the southeastern region, however large smoldering ground fires in the north are becoming more frequent, such as those experienced in Alaska in 2015. From a public health perspective these fires may present additional risk because they tend to occur in places where population may be less informed about protecting themselves during smoke episodes, the smoke exposure at the ground level may be higher and may last longer than similarly sized forest fires, and the relative toxicity of peat fires smoke is likely to differ from smoke from other sources. At least one study showed that particles from the smoldering phase of a fire had differentially higher toxicity on cardiovascular endpoints in animals [39]. Additional research will help establish and support best public health practices during these peatland fire events.

## Conclusion

We used the unique opportunity of a repeat wildfire event in eastern NC to examine whether cardiovascular and respiratory associations with smoke exposure previously observed under similar conditions in 2008 would be replicated. Similar to the 2008 event we found an increased risk of several cardiac and respiratory related visits associated with PM_2.5_ smoke exposure, including for adult upper respiratory infections and respiratory/other chest symptoms, but not for asthma or chronic pulmonary conditions. We also observed an increased risk for the ‘all- cause’ cardiac outcome group which was elevated but not significant during the first fire and for hypertension which was not previously examined. Even though we used the same classification of health outcomes we found limitations in directly comparing the observed associations. Namely, despite the fact the two fires occurred within several miles of each other and burned through similar vegetation, the environmental conditions ascribed spatial and temporal exposure differences in the population at risk. As a result, the 2011 Pains Bay fire produced less severe and more intermittent smoke than the 2008 Evans Road fire. However, the results of this analysis indicate that they both caused similar public health impacts and were both influenced by socioeconomic status [40]. These findings strengthen our understanding of health impacts of peat fire events and the need for improving communication of these impacts to the susceptible populations. The results of our study support the call for additional research on the potential differential toxicities of fuels and the causality of biomass smoke on cardiovascular health effects.

## Additional file

Additional file 1:
**Figure S1.** Percent of population “in poverty” by county (US Census Bureau 2012). **Figure S2.** Comparison of SFS PM_2.5_, satellite imagery and FRM monitor PM_2.5_: May 11, 2011. **Figure S3.** Comparison of cRR (per 10 μg/m^3^ rise in wildfire PM_2.5_) between statistical model adjusted for county-level poverty (used in this study) and an un-adjusted (crude) statistical model. **Figure S4.** Percent change in ED visits and 95 % confidence intervals per 10 μg/m^3^ rise in wildfire PM_2.5_ for adults and stratified by gender. (DOCX 4293 kb)
