# Supplementary material for: Repeating cardiopulmonary health effects in rural North Carolina population during a second large peat wildfire
Source: Environ Health. 2016 Jan 27;15:12. doi: 10.1186/s12940-016-0093-4 (PMC4728755; doi:10.1186/s12940-016-0093-4)

***REPEATING CARDIOPULMONARY HEALTH EFFECTS IN RURAL NORTH CAROLINA POPULATION DURING A SECOND LARGE PEAT WILDFIRE***

Running title: REPLICATING HEALTH EFFECTS OF PEAT WILDFIRE SMOKE

Authors:

Melissa A. Tinling, MSPH, Department of Horticulture, North Carolina State University (mtinlin@ncsu.edu)

J. Jason West, PhD, Environmental Sciences and Engineering, Gillings School of Global Public Health, University of North Carolina, Chapel Hill, NC (jjwest@email.unc.edu)

Wayne E. Cascio, MD, United States Environmental Protection Agency / National Health and Environmental Effects Research Laboratory / Environmental Public Health Division, NC (Cascio.Wayne@epa.gov)

Vasu Kilaru, MS, United States Environmental Protection Agency / National Exposure Research Laboratory / Environmental Sciences Division / Research Triangle Park, NC (vasu.kilaru@epa.gov)

Ana G. Rappold, PhD, United States Environmental Protection Agency / National Health and Environmental Effects Research Laboratory / Environmental Public Health Division, NC (Rappold.Ana@epa.gov)

Corresponding author:

Ana G. Rappold

109 T.W. Alexander Drive

US EPA

Research Triangle Park, NC 27707

919 843 9504

[rappold.ana@epa.gov](mailto:rappold.ana@epa.gov)

The authors declare they have no actual or potential competing financial interests to disclose. This work was conducted as part of the masters’s thesis by Melissa Tinling at the Department of Environmental Science and Engineering at the Gillings School of Global Public Health, University of North Carolina at Chapel Hill. During this time Melissa was employed part-time Program Assistant at the Department of Epidemiology at the North Carolina Division of Public Health in Raleigh, North Carolina.

**Data Attribution and Disclaimers:**

NC DETECT is a statewide public health syndromic surveillance system, funded by the NC Division of Public Health (NC DPH) Federal Public Health Emergency Preparedness Grant and managed through collaboration between NC DPH and UNC-CH Department of Emergency Medicine’s Carolina Center for Health Informatics. The NC DETECT Data Oversight Committee does not take responsibility for the scientific validity or accuracy of methodology, results, statistical analyses, or conclusions presented.

**Disclaimer:** The research described in this article has been reviewed by the National Health and Environmental Effects Research Laboratory, U.S. Environmental Protection Agency, and approved for publication.  Approval does not signify that the contents necessarily reflect the views and policies of the Agency, nor does the mention of trade names of commercial products constitute endorsement or recommendation for use.

Figure S1: Percent of population in poverty by county (US Census Bureau 2012).


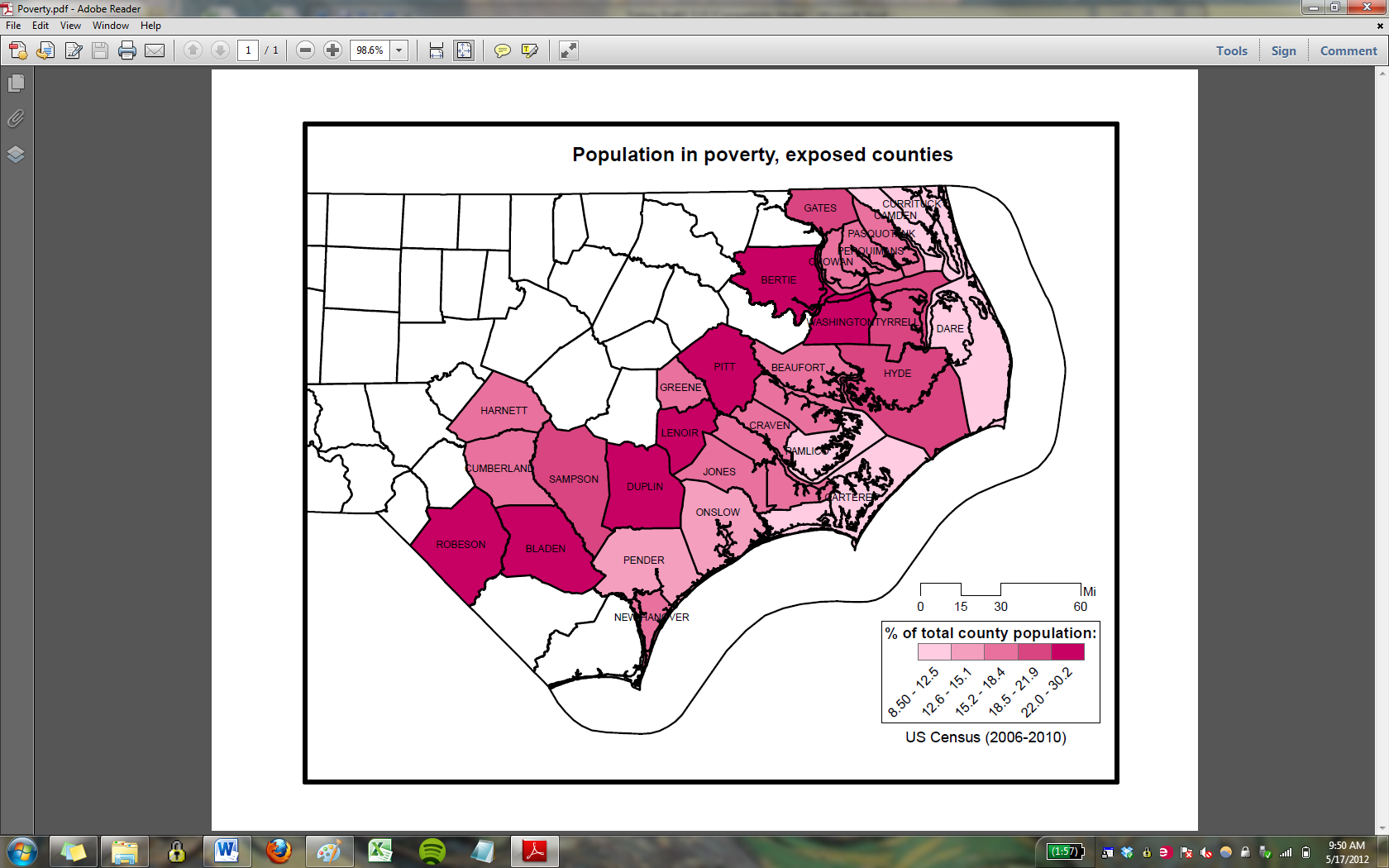


Figure S2: Comparison of SFS PM_2.5_, satellite imagery and FRM monitor PM_2.5_: May 11, 2011.

| SFS HYSPLIT model PM_2.5_ prediction: | 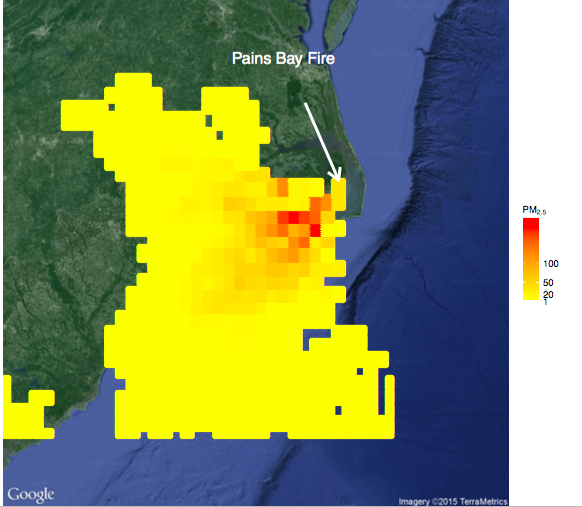 |
| --- | --- |
| Federal Reference Monitor 24-hr PM_2.5_: | 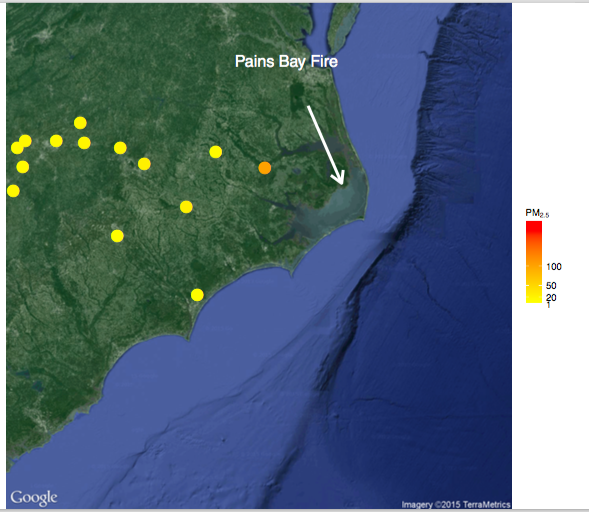 |
| Modis Rapid Response Satellite Imagery: | 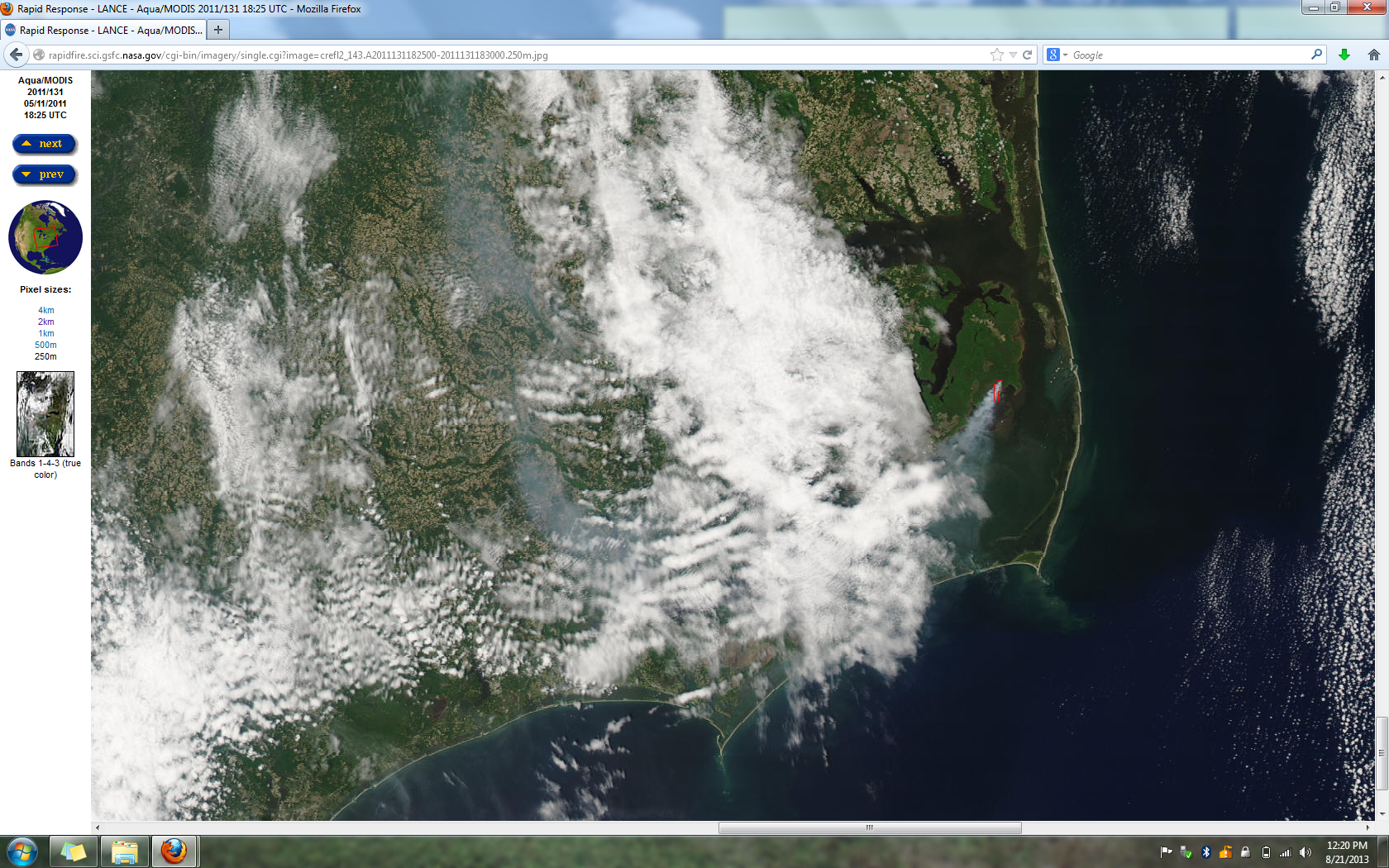 |

Figure S3. Comparison of cRR between statistical model adjusted for county-level poverty (used in this study) and an un-adjusted (crude) statistical model.

Figure S4. Percent change in ED visits and 95% confidence intervals per 10 µg/m^3^ rise in wildfire PM_2.5_ for adults, stratified by gender.


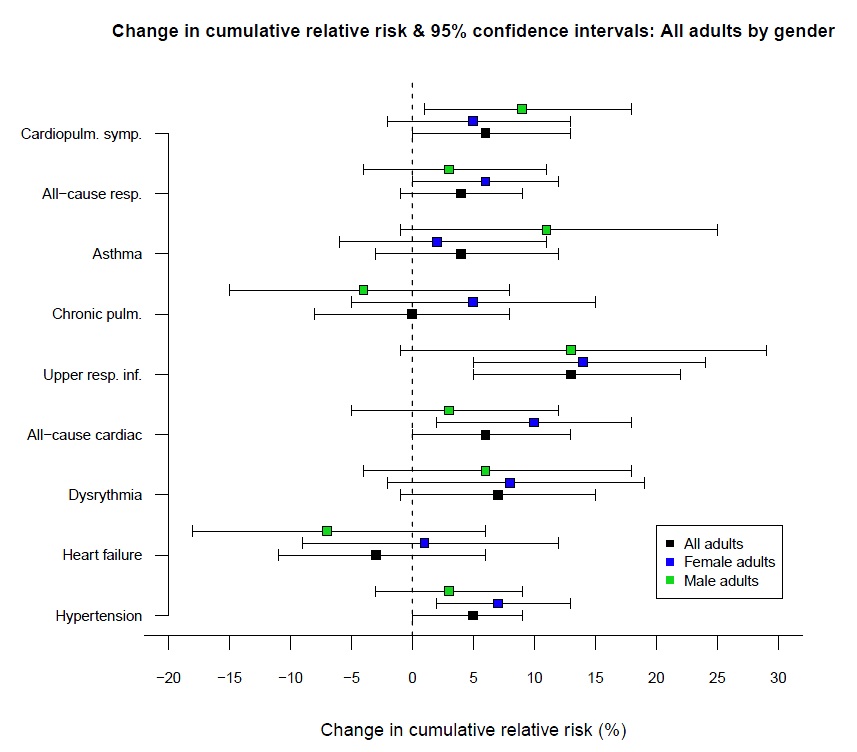

Supplement: Additional file 1: — Figure S1. Percent of population “in poverty” by county (US Census Bureau 2012). Figure S2. Comparison of SFS PM2.5, satellite imagery and FRM monitor PM2.5: May 11, 2011. Figure S3. Comparison of cRR (per 10 μg/m3 rise in wildfire PM2.5) between statistical model adjusted for county-level poverty (used in this study) and an un-adjusted (crude) statistical model. Figure S4. Percent change in ED visits and 95 % confidence intervals per 10 μg/m3 rise in wildfire PM2.5 for adults and stratified by gender. (DOCX 4293 kb) [file 12940_2016_93_MOESM1_ESM.docx]
